# 

**Published:** 1991-03

**Authors:** 


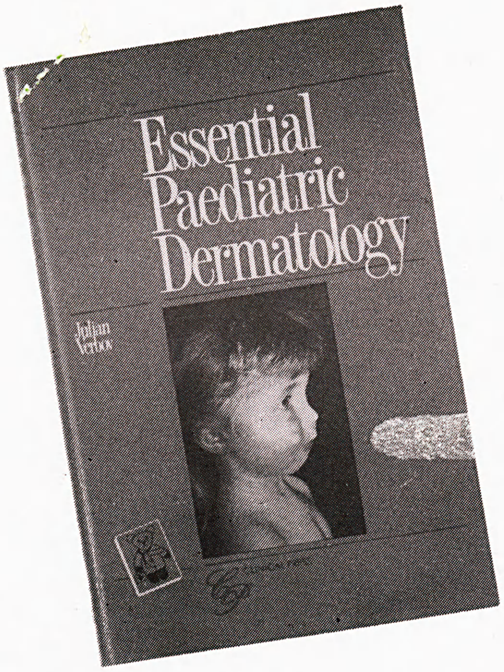

### Essential Paediatric Dermatology

*Julian Verbov, MD., FRCP., FIBiol.,*

Consultant Paediatric Dermatologist,
Royal Liverpool Children's Hospital

**Contents**
The approach to parent and child: The Newborn: Atopic and other dermatitis: Infections and infestations: Napkin area eruptions and erythematosquamous disorders: Hair and nails: Naevi and nodules: Connective-tissue disorders: Vascular disorders and drug eruptions: Genodermatoses: Non-hereditary bullous diseases and mastocytoses: Acne, trauma, light eruptions and pigmentation disorders: A selection of recommended prescribable proprietary preparations: Useful reading: Index

200 pages. Hardcover. 194 illustrations in full colour.
ISBN: 1 85456 000 5. Price £25.00

'This comprehensive and well-illustrated book aims to provide a wider understanding of skin diseases in children both in their diagnosis and management, and would be a useful addition to any practice library.' *Pulse February 4th 1989.*

*'Full marks to Dr. Verbov, who has provided a crisp and lucid text suitable for trainees in medicine, paediatrics, and dermatology. In twelve chapters (starting with The Approach to the Child and Parent) most of the common skin conditions are described and beautifully illustrated in colour. Appendices deal with prescribing in dermatology and offer suggestions for further reading; there is a comprehensive index and each chapter concludes with selected contemporary references.'* **Lancet.**

---

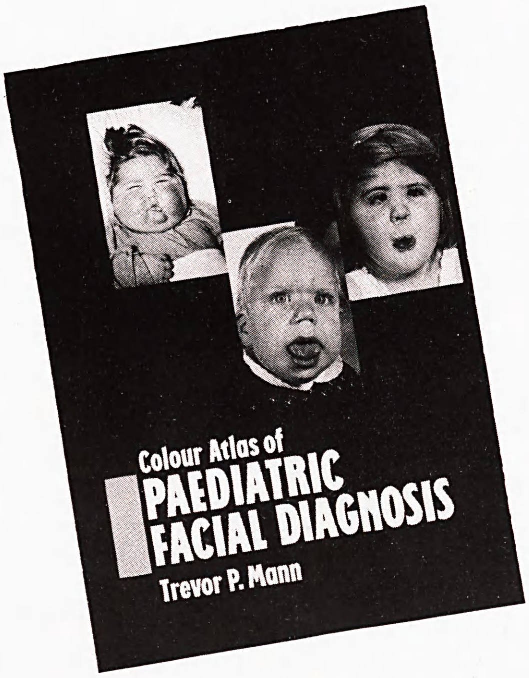

### Colour Atlas of Paediatric Facial Diagnosis

*Trevor P. Mann, MD., FRCP., DCH.,*

Honorary Consultant Physician,
Royal Alexandra Hospital for Sick Children,
Brighton

208 pages. Hardcover. 297mm × 210mm.

425 full colour illustrations.

ISBN: 1 85457 001 3. Price £70.00

This magnificent book demonstrates the methodology of facial diagnosis in children of all ages, including the new born. All common conditions in which facial diagnosis is important are to be found together with many diagnostic rarities. Each topic consists of a concise, informative and scholarly text, supported, where appropriate, by key references, many giving an historical perspective. There are over 400 high quality colour plates with descriptive legends, many of these analysing in some detail the individual features of a face regarded as abnormal or establishing a definitive diagnosis. The importance of recognising subtle expressive changes and 'facial signals' is considered in relation to emotional disorders. Throughout the book are tables listing important diagnostic clues, cranio-facial and otherwise.

*Clinical Press*

Please order from your usual bookseller or direct from Clinical Press Ltd., c/o Gazelle Book Services, Falcon House, Queen Square, Lancaster, LA1 1RN, UK

### WEST OF ENGLAND MEDICAL JOURNAL

**MARCH 1991** **Volume 106 (i)**

Copyright © West of England Medical Journal Ltd 1991.

**Editorial Committee**
Chairman of Editorial Board, Professor John Farndon
Editor— Mr M. G. Wilson
Assistant Editors—Dr Paul Goddard, Dr Alison Round
Secretary—Mr Clive Ward
Members—Dr John Burton, Dr Kenneth Southgate

**Editorial Office**—*The Red House, 40 Coombe Lane, W.O.T., Bristol BS9 2BJ*
*Tel 0272 682320*

**Correspondents**
Dr Andrew Adam (Taunton)
Mr Martin Crosfill (Truro)
Dr Jack Davies (Bristol)
Professor Denis Pereira Gray (Exeter)
Dr Jose Jancar (Bristol)
Dr Michael Oliver (Barnstaple)
Dr Michael Whitfield (Bristol)

**Associated Societies**
Bristol Medico-Chirurgical Society
Clinical Society of Bath
Severn Faculty of the Royal College of General Practitioners
South West of England and Wales Society of Dermatology
South West Obstetricians' and Gynaecologists' Club
South West Orthopaedic Club
South West Paediatric Club
South West Radiologists' Association
South West Urologists' Club
South West Vascular Surgeons
Surgical Club of South West England
Tamar Faculty of the Royal College of General Practitioners
Wessex Association of Clinical Pathologists
West Country Chest Physicians' Club
West Country Physicans' Club

Members of the above organisations receive the journal as part of their membership subscription.
Annual Subscription Rate £25.00 including surface postage.

**Published by**
Clinical Press,
*15 Bucklands Drive,*
*Nailsea,*
*Bristol BS19 2PH*

**Typesetting by**
*SuttonSet, Printing House, Barton Road, Torquay.*

**Printed by**
*The Devonshire Press Ltd, Printing House, Barton Road, Torquay*

**THE WEST OF ENGLAND MEDICAL JOURNAL acknowledges with gratitude the help it receives from NUFFIELD HOSPITALS**

**Contents**

1883–1991 Vols 1–104

**NOTICE TO CONTRIBUTORS**
Contributions are invited especially from West of England authors on a variety of subjects, e.g. Original articles relating to the practice of medicine, reports on the contemporary medical scene, obituaries, historical accounts, recollections of colleagues and events worthy to be remembered and liable to be forgotten. An article of 2,000 words with 2 illustrations will occupy 2 pages and this is a suggested, though by no means an obligatory length. Articles are preferred in the form proposed by the International Committee of Medical Editors (Vancouver System) — pamphlet obtainable from Editor of BMJ, BMA House, Tavistock Square, London, WC1H 9JR. Please send two copies retaining a further copy for your reference. **Articles published in this Journal are listed in Index Medicus.**

REGAINING BLADDER CONTROL

*Second edition*

Clinical Press

Eileen Montgomery, MCSP.,

For those suffering from incontinence on exertion or following pelvic surgery.

This important patient handbook contains a planned series of exercises aimed at restoring control of the bladder. The anatomical causes of incontinence are clearly explained and illustrated. The book also deals with correct breathing and a balanced diet which will not lead to constipation or to being overweight.

Surgeons, doctors and physiotherapists will be able to recommend this booklet to their patients.

28 pages. Soft cover. 11 line drawings. ISBN: 1 85457 003 X

Price inclusive postage and packing £2.50 per copy or £20.00 per pack of 10 copies.

Please order from your usual bookseller or direct from Clinical Press Ltd., c/o Gazelle Book Services, Falcon House, Queen Square, Lancaster, LA1 1RN, UK.

Keith Thompson, MB ChB, FRCGP, DObstRCoG, General Practitioner, London, Examiner for the Diploma in Geriatric Medicine

'Keith Thompson's book should become the keystone of teaching in geriatrics, not only in vocational training for general practice, but also for the growing specialty of hospital and community geriatrics and not only for doctors, but also for nurses, health visitors and social workers.' *Update*

**Contents:**
First Principles. Population and Population Structure. Old People in the Practive. Basic Principles. Attitudes in the Care of the Elderly. Special Difficulties with Older Patients. Practice Organisation. The Team Concept. The Examination. The Investigation. Health Education and Follow up. The Ageing Process. Common Clinical Problems. Specialty Aspects. The Whole Person. Index

*150 pages. 216 x 138 mm. Hardcover. Fully illustrated. ISBN 1 85457 007 2. £12.50*

*Second edition*

**REGAINING BLADDER CONTROL**

Eileen Montgomery, MCSP

This important patient handbook contains a planned series of exercises aimed at restoring control of the bladder. The anatomical causes of incontinence are clearly explained and illustrated. The book also deals with correct breathing and a balanced diet which will not lead to constipation or to being overweight.

*28 pages. Soft cover. 11 line drawings. ISBN : 1 85457 003 X*

*Sold in single copies (£2.50) or in packs of 10 copies. (£20.00)*

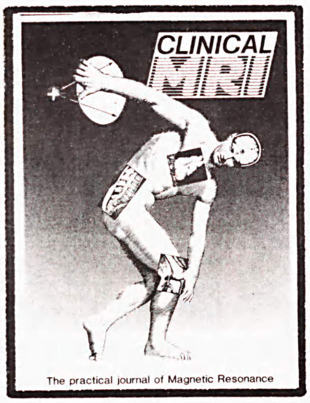

Two issues of *Clinical MRI,*
*The Practical Journal of Magnetic Resonance*
have now been published. Don't miss the next issue of this useful journal. Subscribe now !!!

*UK PRICE: ONLY £ 30*
*PER ANNUM* *from*

CLINICAL PRESS

*************************************

### CLINICAL FILM VIEWING SERIES:

### THE RESPIRATORY SYSTEM

BY PAUL GODDARD MD,FRCR

This book will assist candidates to prepare for the final examination of the FRCR. The book consists of eighty profusely illustrated cases and many lists and flowcharts describing the modern practice of chest radiology. The text is readable and concise.

*This is an invaluable learning book !* *ISBN : 1 85457 014 5* *£ 15.00*

### THE CARDIOVASCULAR SYSTEM

by GEORGE HARTNELL FRCR

"A well-organized presentation of case material which will help residents in radiology and other specialities to assess their knowledge of radiological diagnosis of cardiovascular disease"
A. de Roos MD, European Journal of Radiology

*ISBN: 1 85457 012 9* *£ 15.00*

*COMING SOON : ULTRASOUND by KABALA and SLACK*

*ISBN: 1 85457 013 7* *£ 15.00*

**************************************************************************

*For students of radiology and radiography*

### LECTURE NOTES ON

### THE PHYSICS OF RADIOLOGY

by SUSAN ARMSTRONG FRCR

The principles of radiological physics in a concise and comprehensive format

*ISBN: 1 85457 0102* *£ 15.00*

Please order books from your usual bookseller or direct from Clinical Press Ltd.
c/o Gazelle Book Services Ltd ,Falcon House, Queen Square,Lancaster,LA1 1RN,UK
*Clinical MRI* is available from Redland Green Farm, Redland, Bristol BS6 7HF,UK

